# Translation, cross-cultural adaptation and clinimetric properties of the Brazilian Portuguese version of the Brace Questionnaire

**DOI:** 10.1007/s43390-024-00883-2

**Published:** 2024-04-30

**Authors:** Isabela Pedrosa Fernandes, Marcella Veronnica Pereira Gomes, Rodrigo Mantelatto Andrade, Ariane Verttú Schmidt, Ana Paula Ribeiro, Mauricio Oliveira Magalhães

**Affiliations:** 1 Faculty of Physical and Occupational Therapy, Institute of Health Sciences, Federal University of Pará (UFPA), Belém, Brazil; 2 Post Graduation Program in Human Movement Sciences, Universidade Federal do Pará (UFPA), R. Augusto Corrêa, 01, Belém, PA 66075-110 Brazil; 3 Post Graduation Program in Reabilitation Sciences, University of Sao Paulo (USP), São Paulo, Brazil; 4 Escoliose Brasil Institute, Campinas, Brazil; 5 Laboratório de Biomecânica e Reabilitação Musculoesquelética, Health Science Post-Graduate Department, University Santo Amaro, São Paulo, Brazil

**Keywords:** Brace Questionnaire, Quality of life, Validation, Cross-cultural adaptation, Scoliosis, Brazilian-Portuguese translation

## Abstract

**Purpose:**

To perform a cross-cultural adaptation and validation of the Brazilian-Portuguese versions of the Brace Questionnaire in adolescent idiopathic scoliosis.

**Methods:**

A forward-backward translation process was employed to produce a Brazilian Portuguese version of the Brace Questionnaire, followed by comprehensive cross-cultural adaptation stages. The measurements of internal consistency and test–retest reliability were assessed by Cronbach’s a and intraclass correlation coefficient (ICC), respectively. The Pearson’s correlation coefficient was used to analyze the concurrent validity by comparison with the Scoliosis Research Society-22r questionnaire.

**Results:**

A total of 84 scoliosis patients (age 13.4 ± 2.0 years, thoracic Cobb angle 33.3° ± 13.8°, and lumbar Cobb angle 29.8° ± 14.3°) were included. The Brace Questionnaire showed excellent internal consistency (Cronbach *α* = 0.93) and moderate reliability (ICC = 0.86). The correlations between the Brace Questionnaire and Scoliosis Research Society-22 were *r* = 0.66; *p* = 0.011. In addition, it was found that the Brazilian version of the Brace Questionnaire does not have ceiling and floor effects.

**Conclusions:**

The Brazilian-Portuguese adaptation of the brace questionnaire shows excellent reliability and can be a valid tool for psychometric assessment in adolescent idiopathic scoliosis.

**Supplementary Information:**

The online version contains supplementary material available at 10.1007/s43390-024-00883-2.

## Introduction

Adolescent idiopathic scoliosis (AIS) presents itself as a three-dimensional deformity, altering the physiological structure of the spine [1], and it is more prevalent in patients over the age of 10 years. As the etiology of AIS is unknown, its diagnosis is made through exclusion and the presence of a Cobb angle greater than 10° [2, 3]. Some instruments are available to track progression, of which the scoliometer, which provides an objective measurement of the angle of rotation of the trunk (ART) during anterior flexion, gives a positive sign when the ART ≥ 0 7° [1, 4]. Furthermore, the Adam flexion test can serve as a screening tool in adolescents with idiopathic scoliosis. Other tools used for the diagnosis, such as the Adam flexion test and the image exam, confirm diagnosis and measure the Cobb angle [3, 5, 6].

Worldwide, the prevalence of AIS is approximately 2%, ranging from 1.03 to 6.70% in Brazil; this range is justified by the lack of methodological standardization of studies [5]. In patients with curves between 25° and 45°, conservative treatment associated with the use of a brace is recommended [1]. The brace has the purpose of maintaining the spine growth mechanically aligned and must be worn for a full period of up to 20 h/day [7]. The treatment follow-up and monitoring are essential, due to a series of functional clinical complications observed in adolescents, including moderate impairment on psychological, motor, social, pain, and school environment domains of the quality of life [8]. Thus, quality of life-specific questionnaires can assist in monitoring the condition of these patients and improve the effectiveness of treatment [1].

Some specific questionnaires, including the Quality of Life Profile for Spine Deformities [9] and the Scoliosis Research Society-22r [10, 11], can be used to estimate the quality of life of adolescents with idiopathic scoliosis. The Brace Questionnaire is a specific assessment tool designed by Elias Vasiliadis et al. [12] and is used to assess the quality of life of patients with idiopathic scoliosis undergoing non-surgical (conservative) treatment. This instrument is characterized by eight domains: general health perception, physical functioning, emotional functioning, self-esteem and esthetics, vitality, school activity, body pain, and social functioning [12].

Questionnaires are frequently used by health professionals to determine the condition of their patients or results of treatment; however, most of these instruments are developed in different languages and cultures, demonstrating the need for appropriate adaptation to the target language and culture (15, 16). The original version of the Brace Questionnaire was constructed in Greek [12] and the questionnaire is now available in Italian [13], Polish [14], French [15], Korean [16], Farsi [17], Chinese [18], Dutch [19], Russian [20], and Turkish [21] versions. Clinicians and researchers should use evaluation instruments adapted to their own culture and language to assess the quality of life of adolescents with idiopathic scoliosis in a valid and reliable manner [22].

Thus, the objective of this study was to translate and cross-culturally adapt the Brace Questionnaire to Brazilian-Portuguese and test its measurement properties in a sample of patients with AIS. A secondary objective was to test the measurement properties of this questionnaire in patients with AIS and correlate its score with the Scoliosis Research Society-22r questionnaire (SRS-22) score.

## Methods

### Study design

The use of the original questionnaire was approved by the original authors. The study was approved by the Research Ethics Committee (No. 5.948.047). Data collection was also approved by the administration team, and informed written consent was obtained from each child’s parents.

### Sample

In total, 84 patients were recruited at a reference clinic for the treatment of scoliosis. Inclusion criteria were patients with idiopathic scoliosis, confirmed by imaging examination (Cobb angle above 10°); aged between 10 and 18 years; wearing the brace for more than 3 months, for a minimum of 12 h a day; Cobb angle between 20° and 45°; and types of curvatures: thoracolumbar, thoracic, lumbar, or double. At the assessment moment, all participants wore a thoraco lumbosacral orthosis (TLSO) brace and had no history of surgery.

### Instruments

#### Brace Questionnaire

The Brace Questionnaire [12] consists of 34 questions subdivided into 8 domains: general health perception (items 1 and 2), physical functioning (items 3–9), emotional functioning (items 10–14), self-esteem and esthetics (items 15 and 16), vitality (items 17 and 18), school activity (items 19–21), body pain (items 22–27), and social functioning (items 28–34). The items are formulated to be answered on a 5-category Likert scale of responses: “Always,” “Often,” “Sometimes,” “Rarely,” and “Never.” Each question has a value ranging from 1 to 5, which is multiplied by 20. The final score is the total of all scores divided by 34, ranging from 20 to 100 points, and the higher the score, the better the quality of life. Note: Always = 5, often = 4, sometimes = 3, almost never = 2, and never = 1 for questions 4, 5, 6, 12, 14, 15, 16, and 17, and always = 1, often = 2, sometimes = 3, almost never = 4, and never = 5 for questions 1, 2, 3, 7, 8, 9, 10, 11, 13, 18, 19, 20, 21, 22, 23, 24, 25, 26, 27, 28, 29, 30, 31, 32, 33, and 34.

#### SRS-22r

The Scoliosis Research Society-22r questionnaire covers the following five domains: function/activity (five items); pain (five items); self-image/appearance (five items); mental health (five items); and satisfaction with management (two items). For each item of the domains, the score ranges from 1 (worst) to 5 (best). The total score ranges from 22 to 110 points and the higher the score, the better the quality of life. The Scoliosis Research Society-22r has been shown to display good score distribution, internal consistency, reproducibility, and concurrent validity [23]. The SRS-22 has been successfully translated into Brazilian-Portuguese with acceptable reliability and validity [24].

### Transcultural adaptation

The translation and cultural adaptation process for the Brace Questionnaire followed consensus-based standards for the selection of health measurement instruments (COSMIN) [25] which recommend the use of the Guidelines for the Process of Cross-Cultural Adaptation of Self-Report Measures [26] composed of five parts: initial translation, translation synthesis, back-translation, expert committee review, and test of the pre-final version.

- Step I: Initial translation: A Greek technical translator and a health professional independently translated the original Greek BrQ into Brazilian-Portuguese.
- Step II: Translation synthesis: The differences between the translator and the health professional were discussed between them, using the English version provided by the BrQ author (Dr. Elias Vasiliadis) as support. From the translation synthesis, a common translation was created.
- Step III: Back-translation: A native Greek translator, fluent in Brazilian-Portuguese, back-translated the synthesized version of the questionnaire into the Greek language. The translator involved in this process did not have any health training.
- Step IV: Expert committee review: The Greek and Brazilian-Portuguese versions were reviewed by the committee, consisting of two physiotherapists with clinical and research experience in the area and the translators and researchers involved in the study. This step allowed the resolution of discrepancies, adjustment of inappropriate terms, and verification of equivalence between the versions produced.
- Step V: Test of the pre-final version: In the test phase, 30 patients were asked to complete the questionnaire. After completion of the questionnaire, the participants were asked about their understanding of the BrQ-Br items.

### Procedures

Patients were invited to participate in the study after seeking physical therapy treatment. Confirmation of the diagnosis of idiopathic scoliosis was based on clinical tests and image examination. Both instruments were applied to the participants: the Portuguese-Brazilian version of the Brace Questionnaire (BrQ-Br) and the SRS-22. After 7 days, the BrQ-Br was administered again by telephone. In the initial evaluation, the internal consistency, construct validity, and ceiling and floor effects were measured. In the 7-day evaluation, reliability was tested.

### Statistical analysis

All statistical analyses were guided by SPSS (version 20.0). The normality of the questionnaire data was tested using the Shapiro–Wilk test. The internal consistency was calculated using Cronbach’s alpha to assess the homogeneity of the items in the Brace Questionnaire-BrQ domains, and the appropriate range of alpha values is considered to be between 0.7 and 0.9 [27]. Reliability was determined using the Type 2.1 intraclass correlation coefficient (ICC) with a 95% confidence interval (CI) in the inter-test and retest and intradomain analysis of the BrQ questionnaire. The ICC classification was according to the following parameters: excellent agreement equal to or greater than 0.90, moderate agreement between 0.80 and 0.89, acceptable agreement between 0.71 and 0.79, and worst agreement less than or equal to 0.70 [28]. To analyze the systematic inter-test and retest errors of the measurements of the BrQ questionnaires, the standard error of the measure (SEM) and the standard error of prediction (SEP) were calculated. The SEM was calculated as the ratio between the variability (standard deviation) of the mean differences between the two assessment moments (inter-test and retest) and the √2. The SEP was calculated by the following equation: the product of the variability (SD) of the measure obtained in each test and retest and the √1-ICC2 [27, 29]. To compare the differences between the tests and the retest of the questionnaire, the Student’s *t* test was used and to interpret the magnitude of effect of the difference, the Cohen’s d test was applied. In addition, the Bland–Altman test was applied to verify the agreement between the questionnaire scores: SRS-22 and BrQ (translated version) [30, 31]. The construct validity was performed to verify the correlation between the scores of the questionnaires: SRS-22 and BrQ (translated version) using Spearman’s correlation coefficient. The correlations were according to the following criteria: between 0.0 and 0.30 was considered very weak, between 0.31 and 0.50 weak, between 0.51 and 0.70 moderate, between 0.71 and 0.90 strong, and over 0.91 very strong [32]. The hypothesis of the study was that the score of the BrQ questionnaire (translated version) would present a moderate-to-strong correlation in relation to the SRS-22 questionnaire for adolescents with idiopathic scoliosis. The potential ceiling and floor effects were assessed by calculating the percentage of participants indicating the maximum (ceiling) and minimum (floor) possible scores. These effects were considered to be present if at least 15% of participants scored the maximum or minimum score.

## Results

The translators and back-translators had no difficulty in translating the BrQ; however, the literal translation of some items from Greek to Brazilian-Portuguese could make understanding the questions in the questionnaire difficult or generate double meaning. From the expert committee review and suggestions, the Brace Questionnaire was changed to correct grammatical errors and replace by more suitable terms for the target audience of the questionnaire. The BrQ-Br is presented in Complementary File 1.

In total, 84 adolescents with idiopathic scoliosis were recruited by the research group, including 80 girls and 4 boys. Only one participant did not complete the second stage of the study retest. The duration of the application of the questionnaire was between 10 and 15 min and the participants did not have difficulties completing the questionnaire.

The demographic data of the sample under study are described in Table 1: the mean age of the patients was 13.4 (± 2.0) years, the mean use of the brace was 20.6 (± 2.8) h per day, the mean thoracic Cobb angle was 33.3° (± 13.8°), and the mean lumbar Cobb angle was 29.8° (± 14.3°).

**Table 1:** Anthropometric and clinical characteristics of adolescents with idiopathic scoliosis

Variables	Mean (± SD)	Min.	Max.
Age (years)	13.4 (± 2.0)	10.0	18.0
Body weight (kg)	48.2 (± 8.5)	30.0	72.8
Height (cm)	1.58 (± 12.2)	1.10	2.00
Thoracic Cobb angle (degrees)	33.3 (± 13,8)	0.0	63.0
Lumbar Cobb angle (degrees)	29.8 (± 14.3)	0.0	57.0
Duration of wearing brace (months)	10.4 (± 8.4)	3.0	36.0
Average time spent in brace per day (h)	20.6 (± 2.8)	12.0	23.0

The participants answered the BrQ-Br questionnaires (test and retest), enabling the analysis of internal consistency, by means of the Cronbach’s alpha, and assessment of reliability, using the ICC with a 95% confidence interval, together with the data of mean, standard deviation, ceiling and floor effects of the domains, and final score of the Brace Questionnaire (Table 2). The BrQ-Br presented an excellent Cronbach’s alpha (0.93) and moderate reliability (ICC = 0.86). For all the patients, no floor or ceiling effect was demonstrated in the domains of the BrQ-Br questionnaire.

**Table 2:** Mean, standard deviation, Cronbach’s alpha, intraclass agreement index, comparison before and after 7 days, floor and ceiling effects for each BrQ-Br domain of adolescents with idiopathic scoliosis

BrQ-Br domain	Before Mean (SD)	After Mean (SD)	Cronbach’s alpha	ICC
General health perception	7.3 (1.7)	7.3 (1.6)	0.81	0.69
Physical functioning	27.0 (3.4)	27.5 (4.5)	0.71	0.53
Emotional functioning	18.8 (4.1)	19.5 (4.5)	0.83	0.76
Self-esteem and esthetics	7.6 (1.9)	7.4 (2.0)	0.72	0.63
Vitality	6.8 (1.7)	6.9 (1.8)	0.57	0.59
School activity	13.3 (2.7)	12.9 (2.3)	0.36	0.34
Bodily pain	25.0 (4.0)	24.4 (4.1)	0.82	0.64
Social functioning	26.7 (5.6)	26.6 (6.0)	0.90	0.83
Score total	77.9 (10.8)	77.8 (12.3)	0.93	0.86

The BrQ-Br test and retest reproducibility were considered moderate. The standard error of measurement of the BrQ-Br score was 2.8 in the first test and 0.35 in the retest. The amplitude of the effect observed in the first and second applications of the questionnaire was considered as medium. In addition, a positive and moderate correlation was observed between the BrQ-Br and SRS-22 (*r*; 0.66; *p* < 0.05 (Table 3).

**Table 3:** Test and retest reliability, standard error of measurement and predictive standard error, questionnaire inter-tests, and correlation inter-tests: BrQ-Br and SRS-22r of adolescents with idiopathic scoliosis

Questionnaires	Mean (± SD)	ICC inter-test	SEM	PSE	r
Score BrQ-Br (assessment)	77.9 (± 10.8)	0.65	2.8	3.3	
Score SRS-22r	84.2 (± 12.8)				0.66*

		ICC re-tests			
Score BrQ-Br (after 7 days)	77.8 (± 12.3)	0.54	0.35	0.73	
Score SRS-22r	84.2 (± 12.8)				0.66*

*p < 0.05

**Fig. 1:**
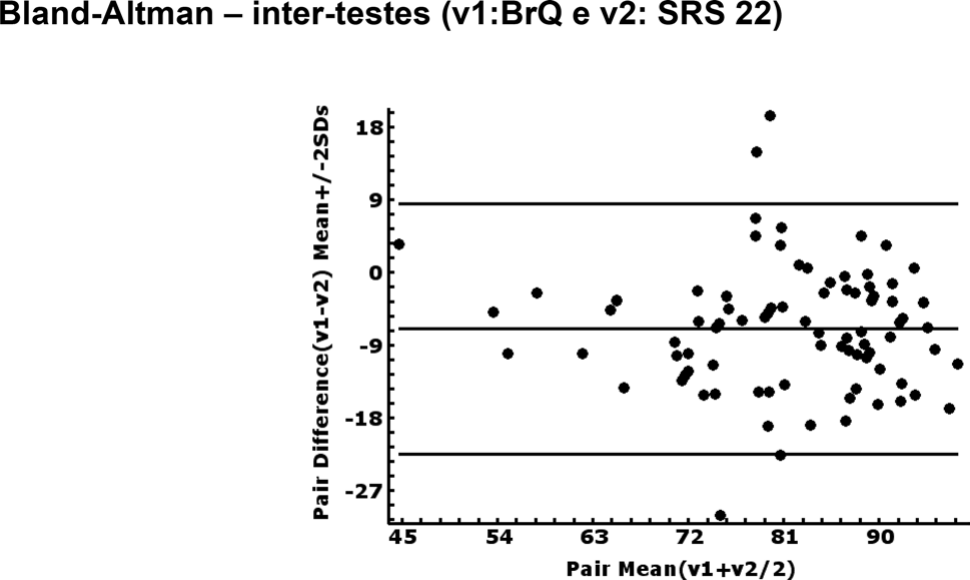
Bland–Altman plot of the differences in the total score on the BrQ between intratests. SRS 22 Brazilian-Portuguese version; SD, standard deviation

**Fig. 2:**
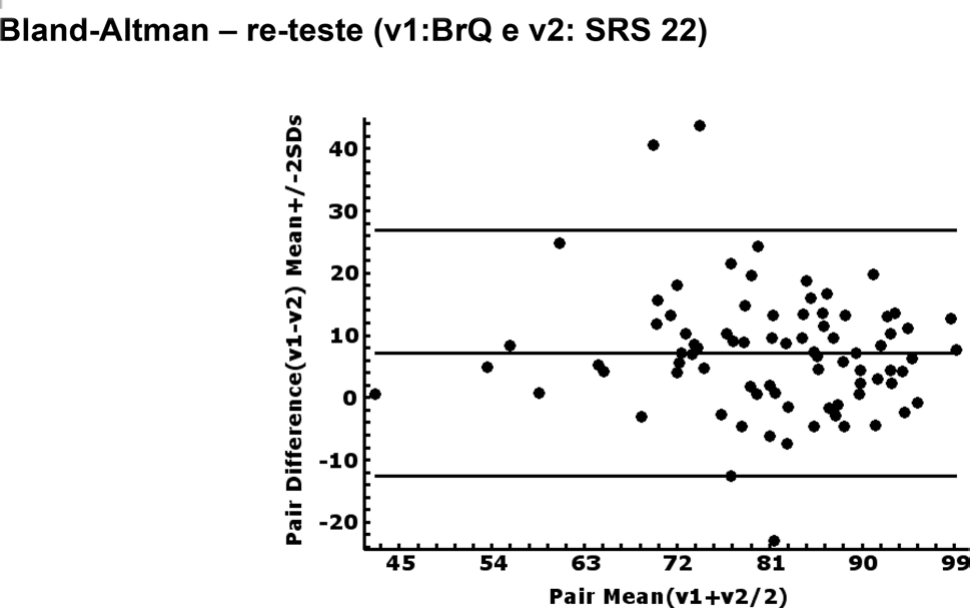
Bland–Altman plot of the differences in the total score on the BrQ between re-test. SRS 22 Brazilian-Portuguese version; SD, standard deviation

In the Bland–Altman analysis of the BrQ-Br and SRS-22 scores, the variations in agreement remained, mostly, within the limits of agreement (Figs. 1, 2).

## Discussion

Adolescent idiopathic scoliosis is one of the most common pathologies affecting the adolescent population [3], with a prevalence rate of approximately 2–3% [1]. The conservative treatment of scoliosis, wearing the brace, and its impact on quality of life are dependent on the patient’s perspective of spinal deformity, as well as on the possible restrictive conditions due to the use of the brace [33, 34]. Several aspects of quality of life can be negatively affected by the prolonged use of the brace (h/day) and the long period of treatment––which is the characteristic of this pathology––can have a major impact on the quality of life of adolescents with scoliosis. In the current study, the mean hours/day of wearing a TLSO vest was 20.6 (± 2.8 h) h, in agreement with the study of Weinsten et al. (2013) and the Sosort 2016 International Consensus Recommendation [1, 35], which can influence the adolescent’s quality of life (QOL). Among the most affected aspects of QoL described in the literature, the physical and psychosocial aspects stand out. These observed impacts are mainly directed at the performance of daily activities due to the perceived physical discomfort when running and playing, among other activities. Regarding the social aspect, the decrease in participation in school activities and reduced social interaction with friends or family are highlighted [8, 36, 37].

Thus, it is suggested that quality of life should be routinely monitored throughout the treatment. Several instruments have been developed to assess quality of life in patients with idiopathic scoliosis, for example the Scoliosis Research Society (SRS) [23] and Bad Sobernheim Stress Questionnaire (BSSQ-K) [38]. However, the BrQ is the first instrument designed to assess the behavioral, physical, and social aspects of a specific population of adolescents with idiopathic scoliosis undergoing conservative treatment with an orthopedic brace.

The process of translating the BrQ into Brazilian-Portuguese was successful and followed COSMIN recommendations [25]. Minor alterations suggested by the expert committee led to better understanding of the items on the BrQ-Br version. The BrQ-Br presented adequate internal consistency (Cronbach’s alpha = 0.93), confirming the homogeneity between the items of the instrument and the importance of all questions for the construct. These findings corroborate with the original BrQ version (Cronbach’s alpha = 0.82) [12], as well as the adaptations in other languages: French (Cronbach’s alpha = 0.85) [15], Polish (Cronbach’s alpha = 0.94) [14], Turkish (Cronbach’s alpha = 0.94) [21], Korean (Cronbach’s alpha = 0.88) [16], Chinese (Cronbach’s alpha = 0.89) [39], Dutch (Cronbach’s alpha = subdomains with a Cronbach’s α ranging between 0.35 for the domain “general health perception and 0.89 for the domain “self-esteem and esthetics) [19], Russian (Cronbach’s alpha = 0.93), and Persian (Cronbach’s alpha = 0.96) [17].

Reliability was measured with an interval of 7 days 1 to 2 weeks between applications of the questionnaire. The intraclass correlation coefficient (ICC = 0.86) found indicates good agreement between the test and retest. In addition, similar results were observed in the study of Chan et al. [39] in the Chinese version (ICC = 0.83), Gür et al. [21] in the Turkish version (ICC = 0.95), Lim et al. [16] in the Korean version (ICC of 0.913), Peeters et al. [19] in the Ducth version (ICC = 0.91), Lein et al. [20] in the Russian version (ICC > 0.9), and Rezaee et al. [17] in the Persian version (ICC = 0.96). Furthermore, the study of Gür et al. [21] guided the translation of the BrQ from the Greek to Turkish version and evaluated the Turkish version as valid (*r* = 0.64, *p* = 0.001). Similar results were found to the Brazilian version (*r* = 0.66; *p* = 0.011), considering a moderate correlation between the BrQ and SRS-22, which supports the hypothesis previously established in our study.

The vitality domain of BRQ-Br showed the lowest score estimated from the responses of the research participants. The items included in this domain ask questions about tiredness and perceived energy disposition with the use of the brace. This estimated effect is dependent on several factors, including the severity of the deformity, the brace model used, and pulmonary function, which can present alterations due to the pathological condition of deformation, as well as the reduced chest mobility with the use of the vest [34, 40]. Verifying this impact on the patient should be considerate as fundamental, to minimize the negative effects on quality of life [41].

To date, this is the first BrQ validation study that presents analyses of SEM, SEP, and the Bland–Altman test to assess agreement. The inclusion of this analysis in future studies is essential to compare and obtain more information about the clinimetric properties of the BrQ in other populations. Given these results, it can be affirmed that the BrQ-Br is easy to apply and could be an important assessment tool to measure the quality of life of adolescents with idiopathic scoliosis.

However, before interpreting the results of the current study, some limitations should be considered. First, the number of subjects included was relatively small. Second, our subjects were recruited at one spine center. Despite this limitation, the extensive analysis of the measurement properties displayed by the BrQ-Br is an important point to be considered in this study.

## Conclusions

The BrQ-Br presented satisfactory validity and reliability for the evaluation of quality of life in AIS using the orthopedic brace and measurement properties similar to the versions produced prior to this research. The validation of this instrument will allow professional researchers and clinicians to conduct an adequate assessment of QOL and management of treatment for AIS.

### Supplementary Information

Below is the link to the electronic supplementary material.

Supplementary file1 (DOCX 30 KB)

## Data Availability

The datasets generated and analyzed during the current study are available from the corresponding author upon reasonable request.
