# Supplementary material for: Translation, cross-cultural adaptation and clinimetric properties of the Brazilian Portuguese version of the Brace Questionnaire
Source: Spine Deform. 2024 Apr 30;12(5):1337–43. doi: 10.1007/s43390-024-00883-2 (PMC11343812; doi:10.1007/s43390-024-00883-2)
Supplement: Supplementary file 1 — Supplementary file1 (DOCX 30 KB) [file 43390_2024_883_MOESM1_ESM.docx]

**Questionário do Colete**

***O questionário a seguir contém perguntas sobre o que você pensa e como se sente em relação à sua saúde. Não é um teste e não existem respostas certas ou erradas.***

- ***Leia cuidadosamente cada pergunta;***
- ***Escolha a resposta que você acha melhor e coloque um X na caixa ao lado:***

| ***Exemplo*** | **Nunca** | **Raramente** | **Às vezes** | **Muitas vezes** | **Sempre** |
| --- | --- | --- | --- | --- | --- |
| ***Durante a semana passada, eu tive disposição para ler*** | **□** | **□** | **□** | ***x*** | **□** |

***Por favor, conte-nos algumas coisas sobre você mesmo:***

**Você é: □ uma menina □ um menino Idade: ……… anos**

**Data.................................................**

| **Durante os três meses anteriores...** | **Nunca** | **Raramente** | **Às vezes** | **Muitas vezes** | **Sempre** |
| --- | --- | --- | --- | --- | --- |
| **1) O colete fez você se sentir doente** | **□** | **□** | **□** | **□** | **□** |
| **2) Você teve medo de que a sua escoliose piorasse** | **□** | **□** | **□** | **□** | **□** |

| **Durante os três meses anteriores...** | **Nunca** | **Raramente** | **Às vezes** | **Muitas vezes** | **Sempre** |
| --- | --- | --- | --- | --- | --- |
| **3) Você se sentiu cansado ao caminhar com o colete** | **□** | **□** | **□** | **□** | **□** |
| **4) Você podia correr com o colete** | **□** | **□** | **□** | **□** | **□** |
| **5) Você vestia o colete sem ajuda** | **□** | **□** | **□** | **□** | **□** |
| **6) Você tirava o colete sem ajuda** | **□** | **□** | **□** | **□** | **□** |
| **7) Você deixou de comer bem quando utilizava o colete** | **□** | **□** | **□** | **□** | **□** |
| **8) Você deixou de dormir bem quando utilizava o colete** | **□** | **□** | **□** | **□** | **□** |
| **9) Você deixou de respirar bem quando utilizava o colete** | **□** | **□** | **□** | **□** | **□** |

| **Durante os três meses anteriores...** | **Nunca** | **Raramente** | **Às vezes** | **Muitas vezes** | **Sempre** |
| --- | --- | --- | --- | --- | --- |
| **10) O colete fez você se sentir nervoso(a)** | **□** | **□** | **□** | **□** | **□** |
| **11) Você se sentiu preocupado(a) por causa do colete** | **□** | **□** | **□** | **□** | **□** |
| **12) Você se sentiu feliz** | **□** | **□** | **□** | **□** | **□** |
| **13) Você acredita que sua vida seria melhor se você não usasse o colete** | **□** | **□** | **□** | **□** | **□** |
| **14) Você acredita que a terapia com o colete lhe ajudou** | **□** | **□** | **□** | **□** | **□** |

| **Durante o mês anterior...** | **Nunca** | **Raramente** | **Às vezes** | **Muitas vezes** | **Sempre** |
| --- | --- | --- | --- | --- | --- |
| **15) Você se sentiu orgulhoso(a) de si mesmo(a)** | **□** | **□** | **□** | **□** | **□** |
| **16) Você se sentiu satisfeito(a) sua aparência** | **□** | **□** | **□** | **□** | **□** |

| **Durante o mês anterior...** | **Nunca** | **Raramente** | **Às vezes** | **Muitas vezes** | **Sempre** |
| --- | --- | --- | --- | --- | --- |
| **17) Você se sentiu forte e cheio(a) de energia** | **□** | **□** | **□** | **□** | **□** |
| **18) Você se sentiu cansado(a) e exausto(a) por causa do colete** | **□** | **□** | **□** | **□** | **□** |

| **Durante o mês anterior...** | **Nunca** | **Raramente** | **Às vezes** | **Muitas vezes** | **Sempre** |
| --- | --- | --- | --- | --- | --- |
| **19) Você tinha dificuldade em suas tarefas da escola por causa do colete** | **□** | **□** | **□** | **□** | **□** |
| **20) Você estava ausente da escola por causa do colete** | **□** | **□** | **□** | **□** | **□** |
| **21) Você sentiu dificuldade para se concentrar na sala de aula** | **□** | **□** | **□** | **□** | **□** |

| **Durante o mês anterior...** | **Nunca** | **Raramente** | **Às vezes** | **Muitas vezes** | **Sempre** |
| --- | --- | --- | --- | --- | --- |
| **22) Você tomava remédios porque estava com dor** | **□** | **□** | **□** | **□** | **□** |
| **23) Você sentiu dor à noite** | **□** | **□** | **□** | **□** | **□** |
| **24) Você sentiu dor quando caminhava** | **□** | **□** | **□** | **□** | **□** |
| **25) Você sentiu dor quando se sentava** | **□** | **□** | **□** | **□** | **□** |
| **26) Você sentiu dor quando subia e descia escadas** | **□** | **□** | **□** | **□** | **□** |
| **27) Você sentiu suas mãos ou seus pés dormentes por causa do colete** | **□** | **□** | **□** | **□** | **□** |

| **Durante o mês anterior...** | **Nunca** | **Raramente** | **Às vezes** | **Muitas vezes** | **Sempre** |
| --- | --- | --- | --- | --- | --- |
| **28) O colete lhe impediu de se encontrar com seus amigos** | **□** | **□** | **□** | **□** | **□** |
| **29) Os seus amigos se lamentavam por causa do problema em sua coluna** | **□** | **□** | **□** | **□** | **□** |
| **30) Você se sentiu diferente dos seus amigos porque usa o colete** | **□** | **□** | **□** | **□** | **□** |
| **31) Você tinha problemas com sua família por causa do colete** | **□** | **□** | **□** | **□** | **□** |
| **32) Você acredita que seu relacionamento com sua família ou seus amigos seria melhor se você não usasse o colete** | **□** | **□** | **□** | **□** | **□** |
| **33) Você ficava em casa porque sentia vergonha do colete** | **□** | **□** | **□** | **□** | **□** |
| **34) Você usava roupas especiais por causa do colete** | **□** | **□** | **□** | **□** | **□** |
